# Service-Specific Heterogeneity in Sepsis Variable Significance and Machine Learning Model Performance: A Stratified Analysis of the BIAlert Cohort

**DOI:** 10.3390/jcm15134904

**Published:** 2026-06-24

**Authors:** Marcio Borges-Sa, Eric Macias-Fassio, Alejandro Delgado, Santiago Salas-Sosa, María Aranda, Antonia Socias, Alberto del Castillo, Andres Giglio

**Affiliations:** 1 Multidisciplinary Sepsis Unit, Son Llatzer University Hospital, 07198 Palma de Mallorca, Spain; marandacorreo@gmail.com (M.A.); asocias@hsll.es (A.S.); adelcastillo@hsll.es (A.d.C.); agiglioj@gmail.com (A.G.); 2 Multidisciplinary Sepsis Group, Health Research Institute of the Balearic Islands (IdISBa), 07120 Palma de Mallorca, Spain; 3 Balearic Islands University, 07122 Palma de Mallorca, Spain; 4 Fundación Código Sepsis, 46003 Valencia, Spain; 5 Instituto de Ingeniería del Conocimiento (IIC), 28049 Madrid, Spain; eric.macias@iic.uam.es (E.M.-F.); aledelmon@outlook.es (A.D.); santiagoenrique.salas@iic.uam.es (S.S.-S.); 6 BiometricsAI, Universidad Autónoma de Madrid, 28049 Madrid, Spain; 7 Critical Care Department, Clínica Las Condes Hospital, Santiago 7591046, Chile; 8 Faculty of Medicine, Finis Terrae University, Santiago 7501015, Chile

**Keywords:** sepsis, machine learning, clinical heterogeneity, hospital department, scoring systems, biomarkers, clinical decision support, Random Forest, artificial intelligence, sepsis phenotypes

## Abstract

**Background/Objectives:** Sepsis detection relies on clinical variables and scoring systems assumed to perform uniformly across hospital settings. However, sepsis phenotype distributions shift between clinical environments, suggesting that variable importance may be setting dependent. This study aimed to quantify service-specific variability in the discriminatory capacity of clinical variables for sepsis detection and to evaluate whether this heterogeneity translates into differential performance of machine learning models compared to traditional clinical scoring systems. **Methods:** This stratified sub-analysis of the BIAlert Sepsis cohort (203,755 patients; 11,864 sepsis episodes, 2014–2018) evaluated 61 structured quantitative variables across nine hospital services (≥90 sepsis episodes each). Within each service, the Mann–Whitney–Wilcoxon test (*p* < 0.01, Holm-corrected) assessed differences between septic and non-septic episodes. Five machine learning models (Random Forest/BIAlert, XGBoost, CatBoost, SVM, Neural Network) and three clinical rules (NEWS, SIRS, qSOFA) were evaluated globally and stratified across four clinical environments. **Results:** The proportion of significant variables ranged from 95.1% in the Emergency Department (58/61) to 37.7% in the Intensive Care Unit (23/61). Lactate was the only universally significant variable (9/9 services). Clinical scoring systems collapsed in Critical Care (qSOFA and NEWS AUC 0.459). BIAlert maintained the highest AUC across all environments (0.975–0.857). The Friedman test confirmed significant differences (χ^2^ = 28.00, *p* < 0.001), with BIAlert achieving a mean rank of 1.0. **Conclusions:** The discriminatory capacity of clinical variables for sepsis detection is not uniform across hospital services. ML models, particularly BIAlert, maintained robust performance where fixed-rule scoring systems failed.

## 1. Introduction

Sepsis is a heterogeneous syndrome whose clinical expression varies substantially across patient subgroups [1]. Over the past decade, phenotyping and endotyping studies have consistently demonstrated that distinct sepsis subpopulations are driven by different clinical and biological variables. Seymour et al. identified four clinical phenotypes with mortality ranging from 5% to 40%, each dominated by different organ dysfunction profiles [2]. Scicluna et al. defined four molecular endotypes from genome-wide blood transcriptomics, with the most lethal subgroup showing immune exhaustion patterns undetectable from clinical variables alone [3]. Sweeney et al. validated three transcriptomic endotypes—inflammopathic, adaptive, and coagulopathic—across 14 international cohorts [4]. Critically, these subgroup distributions are not stable across clinical environments: when the same classification was applied to Intensive Care Units (ICU) populations, phenotype distributions shifted dramatically compared to the original Emergency Department derivation cohorts [5,6]. Whether this setting-dependent redistribution translates into measurable differences in the discriminatory value of individual clinical variables has not been empirically evaluated at the hospital department level.

Traditional scoring systems implicitly assume uniform variable performance, but their behavior contradicts this assumption: qSOFA sensitivity drops to 28.5% in the Emergency Department (ED) and its discrimination collapses to near-random in the ICU, while NEWS outperforms in general wards but was not designed for Critical Care [7,8,9,10]. Meta-analyses have formally identified clinical setting as a significant heterogeneity source [11,12]. Machine learning (ML) models can capture context-dependent relationships across hundreds of variables [13], yet the literature is dominated by single-setting studies (54% ICU-only) and the few hospital-wide models show significant performance variability across environments: the Epic Sepsis Model achieved an AUROC of only 0.63 on external validation [14,15], and even TREWS demonstrated variable impact across hospital types [16]. No study has decomposed this variability to the level of individual clinical variables across hospital departments within a single institution.

This gap is clinically relevant because different departments manage fundamentally different populations: surgical wards see post-operative patients with elevated baseline inflammatory markers, Infectious Diseases units manage chronically infected patients with altered immune profiles, and ICUs concentrate established organ dysfunction. The host response itself varies by infection source [17], and different departments inherently manage different predominant infection types. Consequently, a variable highly discriminative in one service may be uninformative or misleading in another, with direct implications for sepsis surveillance system design.

In a prior study, we developed the BIAlert Sepsis model on a prospectively expert-validated cohort of 203,755 patients, achieving an AUC-ROC of 0.95 with CE marking as a Class IIa medical device [18]. The present study extends this work through a stratified sub-analysis with two objectives: first, to systematically evaluate how the statistical significance of 61 structured clinical variables varies across nine hospital services (≥90 sepsis episodes each), using a formal concordance framework; and second, to compare multiple ML architectures (Random Forest, XGBoost, CatBoost, SVM, Neural Network) against traditional clinical rules (NEWS, SIRS, qSOFA) stratified by service, connecting variable-level heterogeneity to differential model performance.

## 2. Materials and Methods

### 2.1. Sepsis Definition and Outcome Adjudication

Sepsis cases were identified through a real-time prospective validation process led by the Multidisciplinary Sepsis Unit (MSU) throughout the entire study period, as described in detail in the parent publication [18] and the 16-year protocol analysis [19]. The operational criteria for sepsis (“modified Sepsis-2”) required the presence of at least two SIRS criteria (temperature > 38 °C or <36 °C, heart rate > 90 bpm, respiratory rate > 20/min, or white blood cell count > 12,000 or <4000/µL) plus documented evidence of at least one organ dysfunction (hypotension, hypoxemia, oliguria, altered mental status, elevated creatinine, coagulopathy, hyperbilirubinemia, or hyperlactatemia), consistent with severe sepsis or septic shock according to the 2012 Surviving Sepsis Campaign guidelines. Cases flagged through any of six independent detection pathways underwent real-time expert adjudication by the MSU within hours of detection.

Crucially, the outcome label was anchored to the timestamp of MSU validation, and only clinical data from the 48 h preceding this timestamp were used for analysis. This design ensures that the ground truth reflects an audited, expert-validated diagnosis at a defined point in time, rather than retrospective discharge coding, and that the analysis captures the peri-diagnostic window rather than the final clinical picture. While SOFA-component variables (creatinine, bilirubin, platelets, Glasgow Coma Scale (GCS), mean arterial pressure) are included among the 61 structured predictors analyzed, the outcome was not defined by any automated threshold on these variables but by independent bedside clinical adjudication, mitigating the risk of circularity between predictors and outcome.

Although Sepsis-3 (SOFA-based) criteria were available from 2016, modified Sepsis-2 was retained for three reasons: consistency across the entire study period, alignment with the established MSU workflow in place since 2006, and preliminary analysis showing that Sepsis-3 criteria yielded lower sensitivity in this population [18,19]. In the parent study, Sepsis-3 was evaluated as a separate rule-based comparator and demonstrated lower discrimination (AUC-ROC 0.85) than both the modified Sepsis-2 + qSOFA combination (0.90) and the BIAlert model (0.95) [18].

### 2.2. Study Design, Setting, and Parent Cohort

This study is a stratified sub-analysis of the BIAlert Sepsis cohort, fully described in the parent publication [18]. Briefly, the cohort comprises 203,755 patients (218,715 clinical episodes) admitted between January 2014 and December 2018 to Hospital Universitario Son Llàtzer, a 450-bed tertiary center in Palma de Mallorca, Spain. Sepsis cases (*n* = 9301 patients; 11,864 episodes, 5.42%) were prospectively validated in real time by the hospital’s Multidisciplinary Sepsis Unit using modified Sepsis-2 criteria requiring organ dysfunction, as detailed previously [18]. The study was approved by the Ethics and Health Research Committee of the Balearic Community (CEIC-Ib, ID 463721). Informed consent was waived given the retrospective, anonymized design.

### 2.3. Service Stratification

Clinical episodes were stratified at two levels of granularity. For the variable significance and concordance analyses, episodes were stratified by nine individual hospital services with ≥90 sepsis episodes: Emergency Department, General Surgery, Gastroenterology, Internal Medicine, Infectious Diseases, Pulmonology, Oncology, Urology, and ICU. For the model performance evaluation, services were grouped into four clinical environments: Emergency Department (ED), Intensive and Critical Care Units (ICU), Surgical Wards, and Non-Surgical Medical Wards.

### 2.4. Service-Specific Variable Analysis

For each of the 61 structured quantitative variables, the Mann–Whitney–Wilcoxon test compared distributions between septic and non-septic episodes within each service (*p* < 0.01, Holm-corrected). A concordance framework classified each variable as: Positive Concordance (C+) when significant in both service and overall cohort; Negative Concordance (C−) when non-significant in both; and Discordance when significant in one but not the other.

### 2.5. Machine Learning Models

Five ML algorithms were evaluated: Random Forest (BIAlert), XGBoost, CatBoost, Support Vector Machine (SVM), and Multi-Layer Perceptron (Neural Network). These were benchmarked against NEWS, SIRS (≥2 criteria), and qSOFA (≥2 points). Hyperparameters were optimized using Optuna (version 4.8.0) with a TPE sampler over 40 trials maximizing AUC-ROC. The optimal classification threshold was determined via Youden’s Index (J = Sensitivity + Specificity − 1). All analyses were performed in Python 3.13 with scikit-learn 1.8.0, XGBoost 3.2.0, and CatBoost 1.2.10.

### 2.6. Training, Validation, and Evaluation

The dataset was partitioned into training (5/7, ≈71%) and hold-out test (2/7, ≈29%) sets with patient-level independence. Four-fold cross-validation was used for hyperparameter optimization. All models were trained hospital-wide; performance was evaluated globally and by clinical environment. Metrics included AUC-ROC, sensitivity, specificity, precision, NPV, and Brier Score. The Friedman test with Nemenyi post hoc analysis (α = 0.05) was used for model comparison.

## 3. Results

### 3.1. Service-Specific Variable Significance

Of the 61 structured quantitative variables analyzed in the overall cohort, 54 (88.5%) were statistically significant. Seven were non-significant: Glasgow Coma Scale (*p* = 0.23), mean arterial pressure (*p* = 0.20), bowel movements (*p* = 0.31), pain scale (*p* = 0.33), blood LDH (*p* = 0.724), platelets (*p* = 0.22), and plateletcrit (*p* = 0.26).

**Figure 1:**
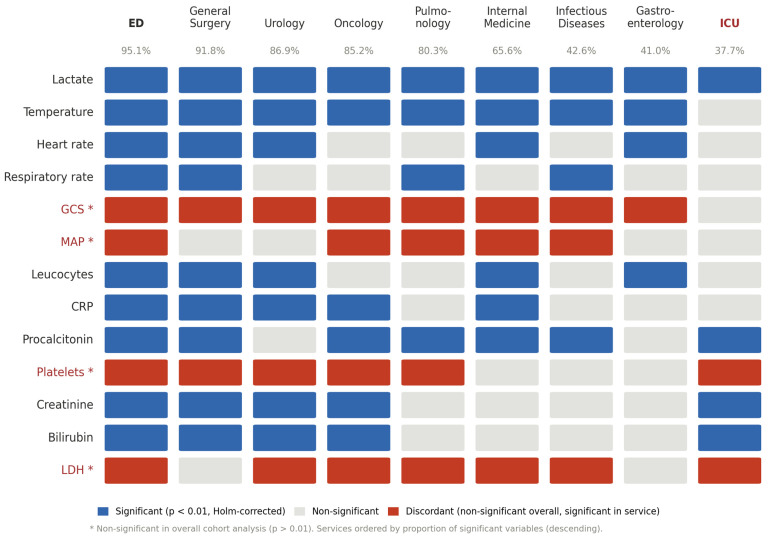
Service-specific significance of selected clinical variables for sepsis detection across nine hospital services. Services ordered by proportion of significant variables (descending). * Variables non-significant in overall cohort analysis. Abbreviations: ED: Emergency Department. ICU: Intensive Care Unit.

When stratified by individual service, the proportion of significant variables ranged from 95.1% in the ED (58/61) to 37.7% in the ICU (23/61) (Table 1, Figure 1). General Surgery (56/61, 91.8%), Urology (53/61, 86.9%), and Oncology (52/61, 85.2%) showed proportions similar to the overall cohort. Gastroenterology (25/61, 41.0%) and Infectious Diseases (26/61, 42.6%) showed proportions comparable to the ICU.

### 3.2. Concordance Analysis

#### 3.2.1. Universally Concordant Variables

Plasma lactate was the only variable that maintained statistical significance across all nine services (C+ = 9/9). Temperature, bicarbonate, and FiO_2_ were significant in 8 of 9 services (C+ = 8/9). For temperature, the ICU was the sole exception.

#### 3.2.2. Variables Significant Overall but Discordant Across Services

Several variables significant in the overall cohort did not reach significance in specific services. C-reactive protein was non-significant in Pulmonology, Gastroenterology, Infectious Diseases and the ICU (C+ = 5/9). Procalcitonin was non-significant in Urology and Gastroenterology. Creatinine was non-significant in Gastroenterology, Internal Medicine, Infectious Diseases, and Pulmonology (C+ = 5/9). Leucocyte count and absolute neutrophils were non-significant in Infectious Diseases, Pulmonology, Oncology, and the ICU (C+ = 5/9). Age was significant in only 2 of 9 services.

#### 3.2.3. Variables Non-Significant Overall but Significant in Specific Services

Of the seven variables non-significant in the overall cohort, six demonstrated discordant behavior. Glasgow Coma Scale was significant in 8 of 9 services (all except the ICU). Mean arterial pressure was significant in 5 of 9 services. Platelets in 6 of 9 services. LDH was significant in 7 of 9 services despite an overall *p*-value of 0.724.

Among the nine services, the ICU had the highest rate of Discordance with the overall cohort. Variables significant overall but non-significant in the ICU included: age, heart rate, respiratory rate, oxygen saturation, blood pressure, leucocytes, neutrophils, lymphocytes, C-reactive protein, albumin, cholesterol, glucose, and multiple hematological parameters.

### 3.3. Global Model Performance

The Friedman test indicated significant differences among models (χ^2^ = 28.00, *p* < 0.001). Mean rankings confirmed BIAlert as top-performing (mean rank 1.0), followed by XGBoost and CatBoost (2.5 each), SVM (4.0), and Neural Network (5.0). Clinical rules ranked lowest: SIRS (6.0), NEWS (7.0), qSOFA (8.0). (Table 2).

### 3.4. Service-Stratified Model Performance

**Figure 2:**
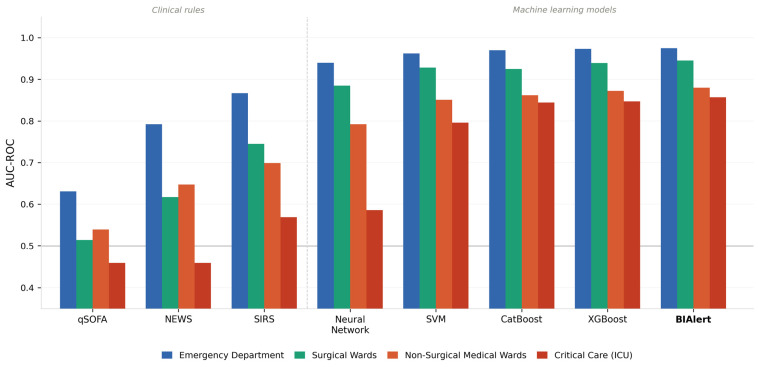
AUC-ROC of clinical scoring systems and machine learning models stratified by clinical environment. The dashed vertical line separates clinical rules from ML models. The solid horizontal line indicates random chance discrimination (AUC = 0.5).

Table 3 and Figure 2 present model performance stratified by clinical environment. Bold marks the best-performing model, BIAlert.

#### 3.4.1. Emergency Department

BIAlert achieved an AUC of 0.975 ± 0.001, followed by XGBoost (0.973 ± 0.001), CatBoost (0.970 ± 0.001), and SVM (0.962 ± 0.003). Among clinical rules, SIRS reached 0.867 ± 0.002, NEWS 0.792 ± 0.012, and qSOFA 0.631 ± 0.007.

#### 3.4.2. Critical Care

All models showed reduced performance. BIAlert achieved 0.857 ± 0.061. XGBoost (0.847 ± 0.065) and CatBoost (0.844 ± 0.065) showed comparable results. SVM maintained sensitivity (0.883 ± 0.070) with low specificity (0.460 ± 0.173). The Neural Network achieved 0.586 ± 0.121. Clinical rules: qSOFA 0.459 ± 0.057, NEWS 0.459 ± 0.133, SIRS 0.569 ± 0.093.

#### 3.4.3. Surgical Wards

BIAlert achieved 0.945 ± 0.004, followed by XGBoost (0.939 ± 0.009), SVM (0.928 ± 0.009), and CatBoost (0.925 ± 0.009). Clinical rule AUCs ranged from 0.514 ± 0.006 (qSOFA) to 0.745 ± 0.017 (SIRS).

#### 3.4.4. Non-Surgical Medical Wards

BIAlert achieved 0.880 ± 0.009. XGBoost (0.872 ± 0.006), CatBoost (0.862 ± 0.006), and SVM (0.851 ± 0.001) showed reduced performance. Clinical rule AUCs ranged from 0.539 ± 0.006 (qSOFA) to 0.699 ± 0.014 (SIRS).

### 3.5. Model Calibration

**Figure 3:**
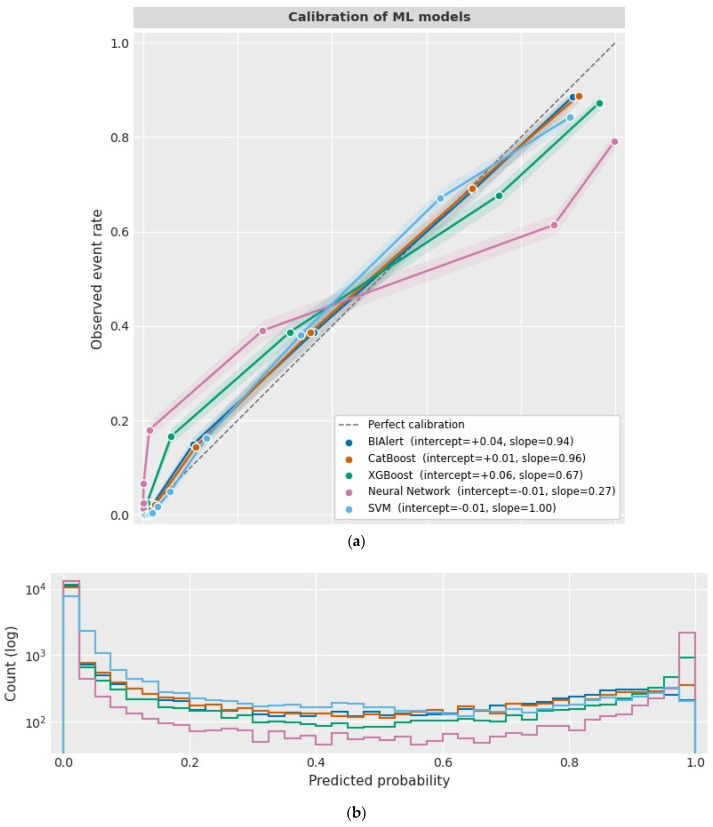
Calibration of the five machine learning models. The top panel (**a**) compares each model’s predicted probability of sepsis with the actual event rate observed in the data; points close to the dashed diagonal indicate well-calibrated predictions, and the shaded bands show 95% confidence intervals. The intercept and slope in the legend summarize overall calibration (perfect calibration corresponds to an intercept of 0 and a slope of 1). The bottom panel (**b**) shows how predicted probabilities are distributed for each model.

Beyond discrimination, calibration was assessed for the five machine learning models (Figure 3). Three models showed excellent calibration, with slopes close to the ideal value of 1 and intercepts near 0: SVM (slope 1.00, intercept −0.01), CatBoost (slope 0.96, intercept +0.01), and BIAlert (slope 0.94, intercept +0.04). XGBoost showed moderate miscalibration (slope 0.67, intercept +0.06), tending to overestimate risk at higher predicted probabilities. The Neural Network was poorly calibrated (slope 0.27, intercept −0.01), with predicted probabilities compressed toward the middle of the range and substantial deviation from the diagonal. Notably, the models with the strongest discrimination were also among the best calibrated, whereas the Neural Network ranked lowest on both dimensions, reinforcing its limited suitability for this task. The probability distributions (Figure 3, lower panel) confirmed that the well-calibrated models produced confident predictions concentrated near 0 and 1, while the Neural Network output was concentrated in the intermediate range.

## 4. Discussion

In this analysis, the statistical association between clinical variables and sepsis varied considerably across hospital services within a single institution, from 95.1% of variables reaching significance in the Emergency Department to 37.7% in the ICU. Several variables in routine use for sepsis identification, such as CRP, leucocytes, and creatinine, did not separate septic from non-septic episodes in particular services. Variables that were not significant in the pooled cohort, including GCS, MAP, platelets, and LDH, did reach significance in individual services. Lactate was the only variable consistently associated with sepsis across all nine services examined. This variability at the level of individual predictors was mirrored at the level of the models: the fixed-rule scores lost discrimination in the settings where their component variables weakened, while the machine learning models held a more stable performance across environments.

A distinguishing feature of this work is the cohort on which the model rests. Most sepsis prediction models are developed on general Critical Care databases such as MIMIC or eICU. BIAlert was instead built on a hospital-wide cohort in which every sepsis event was validated in real time by a multidisciplinary expert unit [18,19]. The difference matters. Models that draw their labels from retrospective discharge coding inherit familiar biases: cases are misclassified, administrative labelling differs between institutions, and the diagnosis is tied to the final clinical picture rather than to the moment of onset [18]. Anchoring the reference instead to the audited timestamp of the event, and using only the data available beforehand, ties the analysis to the peri-diagnostic window and keeps it independent of how the episode was eventually coded or where the patient was discharged from. This is largely what makes the service-stratified reading interpretable, since the heterogeneity it reveals is more plausibly a property of how sepsis presents in different settings than an artefact of classification.

Differences across services look less like a shortcoming and more like a sign of robustness. A hospital-wide model is most useful when its performance survives being taken apart service by service, and BIAlert retained discrimination well above the fixed-rule systems, even where individual variables lost their association. A model that performed well only in aggregate, or only in the setting where it was trained, would give little reassurance about its behavior elsewhere in the same hospital. That BIAlert kept the highest discrimination in every environment despite clear shifts in which variables mattered suggests it has captured a representation of sepsis that travels reasonably well across the heterogeneity of hospital care. Calibration pointed in the same direction: the model ranked first in discrimination and among the best in calibration (slope 0.94, intercept +0.04), which suggests its predicted probabilities can be read as risk estimates rather than as bare rankings.

These observations sit alongside an idea that the phenotyping literature has implied but, to our knowledge, not tested directly at the department level. Seymour et al. described four sepsis phenotypes, each driven by a different set of variables [2], and van Amstel et al. showed that the distribution of those phenotypes shifts markedly between ED and ICU populations, with the α phenotype falling from 33% to between 1 and 6% in ICU cohorts [5,6]. Our concordance analysis adds an empirical step at the service level, suggesting that the importance of a variable depends on the setting and that this dependence can be quantified. The loss of CRP significance in Infectious Diseases and the ICU fits what is known about populations with a high baseline inflammatory burden [17], and the non-significance of leucocytes in Oncology is plausibly related to chemotherapy-induced confounding. GCS is a useful illustration: not significant in the pooled cohort yet significant in most individual services, a pattern in which aggregation across mixed populations can hide an association that holds locally, with consequences for how features are selected in hospital-wide models.

The performance patterns are consistent with earlier work on the variability of scoring systems. The collapse of qSOFA in Critical Care echoes the findings of Raith et al. [20] and Churpek et al. [8], and our data suggest that the same gradient affects the machine learning models, though to a much smaller degree. The contrast between SVM and BIAlert in Critical Care is informative about the usual trade-off: SVM held its sensitivity (0.883) at the expense of specificity (0.460), whereas BIAlert sat at a more balanced point (0.845 and 0.668). Context-dependent degradation has been reported in external validations as well. The Epic Sepsis Model reached an AUROC of only 0.63 outside its development setting [14], with performance that tracked hospital acuity [15], which suggests that part of this degradation may reflect not only differences between institutions but also the intra-institutional heterogeneity across services that we describe here. Most predictive models in this field are built around a single concept, such as bacteremia [21,22], or around a single setting, most often the Emergency Department [23], where they can reach strong performance. The value of the present model lies elsewhere: it was designed hospital-wide, and its discrimination and calibration hold up as it moves across services rather than being tuned to one target or one environment.

Service-level performance must be read against how patients reach each department. Routing pre-selects for the dominant presenting problem, so on non-emergency wards sepsis is more often a complication that develops during admission than the reason for it. This does not narrow the model to the Emergency Department. Sepsis is frequently not an Emergency Department event: in our hospital 46% of protocol activations arose elsewhere, 34.2% in the ICU and 11.8% on general wards, and those patients died more often than ED patients (24.8% in the ICU and 13.5% on wards, against 10.4%) [19]. A tool confined to the Emergency Department would miss precisely the patients at greatest risk. Weaker discrimination in a given service is also not the same as no use. In the ICU, the setting with the fewest significant variables, BIAlert held an AUC of 0.857, well above the fixed-rule comparators (qSOFA and NEWS at 0.459), and the task there is less the initial diagnosis than the timely recognition of deterioration in admitted patients, where hospital-wide surveillance has shown value [16,19]. Because the model is tied to the audited, peri-diagnostic timestamp of each event rather than to the admitting diagnosis or the final disposition, whether sepsis was the reason for admission or a later complication does not bear on the analysis. Performance and the variables that matter shift with the setting, which is itself the argument for a context-adaptive model over a single fixed threshold applied hospital-wide.

What this analysis does not address is deployment. Real-time implementation, the shape of the alerts, the false-positive burden, and the effect on patient outcomes belong to a separate prospective implementation study now underway, and risk-stratified confirmatory testing downstream of the model fits naturally within that work. Some limitations follow from the design, which is a sub-analysis of a single-center retrospective cohort, so external multi-center validation will be needed. The hospital does not house certain specialties, in particular cardiac surgery and neurosurgery, whose case-mix might show different patterns. Grouping services into four environments for the performance analysis may obscure variation within each group, and the variable analysis was limited to the 61 structured predictors. The non-significance of some variables in the pooled cohort, GCS and MAP among them, may partly reflect limited data, for example GCS was recorded in 14.4% which leaves less power for those comparisons whatever their underlying association, but reflects the reality of variable evaluations across different hospital settings. On case definition, the study used modified Sepsis-2 criteria requiring organ dysfunction [19]. Sepsis-3 is not in fact the universal reference: in the European Sepsis Care Survey, 45.4% of 1023 hospitals used Sepsis-3, 24.5% used Sepsis-1, and 23.2% used combined definitions [24], and our approach is consistent with that last group and with the operational standard our institution has kept since 2006. Sepsis-3-based diagnosis and detection have been evaluated in another manuscript showing lower performance as an early sepsis detection tool [18]. A natural next step would be to ask whether service-specific recalibration, adjusting feature weights or thresholds by department, recovers performance where concordance is low, as the transfer learning literature would suggest [25]. It also remains open whether these differences in detection translate into differences in clinical outcome.

## 5. Conclusions

This study shows that the discriminatory capacity of clinical variables for sepsis detection is not uniform across hospital services. Of 61 structured variables, the proportion reaching statistical significance ranged from 95.1% in the Emergency Department to 37.7% in the ICU, with seven variables non-significant overall, achieving significance in most individual services. ML models, particularly BIAlert, maintained robust performance across clinical environments where fixed-rule scoring systems failed. These findings support that hospital-wide sepsis detection models should account for service-level variability and that ML architectures offer a structural advantage over fixed-rule systems in heterogeneous clinical environments.

## Figures and Tables

**Table 1:** Number of structured quantitative variables significantly associated with sepsis (*p* < 0.01, Holm-corrected) by hospital service.

Service	Significant Variables (*n*/61)	%
Emergency Department	58	95.1
General Surgery	56	91.8
Urology	53	86.9
Oncology	52	85.2
Pulmonology	49	80.3
Internal Medicine	40	65.6
Infectious Diseases	26	42.6
Gastroenterology	25	41.0
Intensive Care Unit	23	37.7
Overall Cohort	54	88.5

**Table 2:** Performance metrics for sepsis detection models and clinical rules (mean ± SD).

Model	AUC	Sens	Spec	Prec	NPV	Brier
NEWS	0.752 ± 0.011	0.495 ± 0.015	0.878 ± 0.005	0.524 ± 0.011	0.865 ± 0.003	0.145 ± 0.003
NEWS CI	(0.743–0.760)	(0.479–0.509)	(0.873–0.883)	(0.510–0.537)	(0.861–0.869)	(0.144–0.148)
SIRS	0.816 ± 0.005	0.811 ± 0.008	0.772 ± 0.004	0.491 ± 0.005	0.938 ± 0.002	0.147 ± 0.002
SIRS CI	(0.809–0.823)	(0.799–0.823)	(0.765–0.779)	(0.483–0.500)	(0.934–0.942)	(0.145–0.149)
qSOFA	0.586 ± 0.005	0.209 ± 0.011	0.963 ± 0.002	0.608 ± 0.013	0.818 ± 0.002	0.198 ± 0.036
qSOFA CI	(0.580–0.593)	(0.198–0.222)	(0.960–0.966)	(0.583–0.632)	(0.816–0.821)	(0.197–0.199)
**BIAlert**	**0.957 ± 0.001**	**0.836 ± 0.019**	**0.916 ± 0.008**	**0.730 ± 0.015**	**0.954 ± 0.005**	**0.069 ± 0.001**
**BIAlert** CI	**(0.955–0.960)**	**(0.825–0.847)**	**(0.912–0.920)**	**(0.719–0.741)**	**(0.951–0.957)**	**(0.066–0.071)**
XGBoost	0.954 ± 0.001	0.788 ± 0.013	0.926 ± 0.006	0.744 ± 0.014	0.942 ± 0.003	0.075 ± 0.001
XGBoost CI	(0.952–0.957)	(0.776–0.801)	(0.922–0.931)	(0.733–0.756)	(0.939–0.945)	(0.073–0.078)
CatBoost	0.949 ± 0.001	0.767 ± 0.013	0.916 ± 0.006	0.740 ± 0.014	0.935 ± 0.003	0.085 ± 0.001
CatBoost CI	(0.947, 0.951)	(0.742, 0.792)	(0.904, 0.928)	(0.713, 0.767)	(0.929, 0.941)	(0.083, 0.087)
SVM	0.941 ± 0.002	0.904 ± 0.003	0.847 ± 0.005	0.616 ± 0.007	0.970 ± 0.001	0.080 ± 0.001
SVM CI	(0.938–0.944)	(0.895–0.913)	(0.841–0.852)	(0.607–0.624)	(0.968–0.973)	(0.077–0.082)
Neural Net	0.909 ± 0.007	0.704 ± 0.015	0.910 ± 0.008	0.679 ± 0.020	0.919 ± 0.004	0.112 ± 0.005
Neural Net CI	(0.905–0.914)	(0.690–0.719)	(0.905–0.914)	(0.668–0.693)	(0.915–0.922)	(0.107–0.114)

Bold denotes the BIAlert sepsis model, the best-performing model in this evaluation.

**Table 3:** Performance metrics by clinical environment (mean).

Model	Environment	AUC	Sens	Spec	Prec	NPV	Brier
NEWS	Emergency Dept	0.792 (0.782–0.803)	0.526 (0.505–0.546)	0.917 (0.912–0.922)	0.555 (0.536–0.573)	0.907 (0.904–0.911)	0.116 (0.114–0.120)
	Critical Care	0.459 (0.317–0.592)	0.257 (0.146–0.368)	0.689 (0.462–0.871)	0.688 (0.514–0.842)	0.249 (0.184–0.304)	0.347 (0.273–0.398)
	Surgical Wards	0.617 (0.589–0.647)	0.254 (0.218–0.292)	0.883 (0.867–0.899)	0.397 (0.348–0.447)	0.796 (0.788–0.805)	0.186 (0.181–0.201)
	Non-Surg Med	0.647	0.541	0.698	0.504	0.729	0.220
SIRS	Emergency Dept	0.867 (0.858–0.876)	0.865 (0.851–0.880)	0.827 (0.820–0.833)	0.496 (0.485–0.506)	0.969 (0.966–0.972)	0.126 (0.124–0.129)
	Critical Care	0.569 (0.444–0.686)	0.481 (0.361–0.608)	0.576 (0.349–0.759)	0.773 (0.664–0.876)	0.286 (0.199–0.378)	0.270 (0.190–0.302)
	Surgical Wards	0.745 (0.721–0.770)	0.693 (0.653–0.735)	0.759 (0.738–0.781)	0.465 (0.440–0.493)	0.890 (0.877–0.903)	0.182 (0.176–0.189)
	Non-Surg Med	0.699 (0.684–0.715)	0.783 (0.760–0.804)	0.533 (0.514–0.552)	0.487 (0.476–0.500)	0.813 (0.797–0.829)	0.210 (0.204–0.215)
qSOFA	Emergency Dept	0.631 (0.621–0.642)	0.299 (0.279–0.319)	0.964 (0.960–0.967)	0.619 (0.592–0.645)	0.875 (0.871–0.878)	0.184 (0.182–0.186)
	Critical Care	0.459 (0.383–0.513)	0.030 (0.002–0.077)	0.887 (0.751–0.978)	0.500 (0.100–0.900)	0.247 (0.215–0.268)	0.283 (0.262–0.312)
	Surgical Wards	0.514 (0.506–0.522)	0.032 (0.017–0.049)	0.995 (0.991–0.998)	0.701 (0.495–0.889)	0.772 (0.769–0.775)	0.251 (0.249–0.253)
	Non-Surg Med	0.539 (0.529–0.549)	0.136 (0.118–0.154)	0.942 (0.933–0.951)	0.571 (0.523–0.619)	0.659 (0.654–0.664)	0.257 (0.254–0.298)
**BIAlert**	**Emergency Dept**	**0.975 (0.973–0.978)**	**0.864 (0.850–0.879)**	**0.945 (0.941–0.949)**	**0.757** **(0.742–0.771)**	**0.973** **(0.971–0.976)**	**0.047 (0.045–0.049)**
	**Critical Care**	**0.857** **(0.745–0.945)**	**0.845** **(0.750–0.925)**	**0.668** **(0.441–0.850)**	**0.875** **(0.812–0.940)**	**0.605** **(0.455–0.784)**	**0.161 (0.117–0.214)**
	**Surgical Wards**	**0.945** **(0.935–0.954)**	**0.833** **(0.798–0.867)**	**0.897** **(0.882–0.913)**	**0.712** **(0.681–0.744)**	**0.948** **(0.938–0.958)**	**0.081** **(0.072–0.089)**
	**Non-Surg Med**	**0.880** **(0.869–0.891)**	**0.792** **(0.770–0.814)**	**0.795** **(0.779–0.810)**	**0.687** **(0.668–0.703)**	**0.871** **(0.860–0.884)**	**0.137** **(0.130–0.144)**
CatBoost	Emergency Dept	0.970 (0.967–0.972)	0.815 (0.800–0.830)	0.945 (0.941–0.949)	0.769 (0.754–0.784)	0.945 (0.942–0.948)	0.051 (0.048–0.053)
	Critical Care	0.844 (0.725–0.942)	0.719 (0.624–0.799)	0.734 (0.507–0.916)	0.853 (0.793–0.917)	0.468 (0.306–0.642)	0.230 (0.182–0.286)
	Surgical Wards	0.925 (0.916–0.934)	0.737 (0.701–0.771)	0.903 (0.889–0.917)	0.695 (0.666–0.728)	0.911 (0.900–0.921)	0.189 (0.181–0.197)
	Non-Surg Med	0.862 (0.851–0.873)	0.729 (0.706–0.751)	0.800 (0.784–0.815)	0.692 (0.673–0.709)	0.827 (0.816–0.840)	0.159 (0.151–0.166)
Neural Net	Emergency Dept	0.940 (0.935–0.945)	0.736 (0.717–0.755)	0.941 (0.937–0.946)	0.711 (0.696–0.726)	0.948 (0.945–0.952)	0.077 (0.073–0.081)
	Critical Care	0.586 (0.459–0.701)	0.783 (0.672–0.878)	0.474 (0.291–0.701)	0.809 (0.749–0.872)	0.417 (0.236–0.592)	0.318 (0.236–0.412)
	Surgical Wards	0.885 (0.867–0.903)	0.680 (0.642–0.720)	0.893 (0.880–0.907)	0.659 (0.626–0.694)	0.903 (0.893–0.915)	0.129 (0.117–0.141)
	Non-Surg Med	0.792 (0.777–0.807)	0.658 (0.634–0.683)	0.782 (0.765–0.799)	0.631 (0.612–0.652)	0.802 (0.790–0.814)	0.223 (0.212–0.235)
SVM	Emergency Dept	0.962 (0.959–0.965)	0.919 (0.908–0.930)	0.896 (0.890–0.901)	0.635 (0.621–0.648)	0.982 (0.980–0.985)	0.055 (0.053–0.058)
	Critical Care	0.796 (0.670–0.890)	0.883 (0.788–0.962)	0.460 (0.278–0.687)	0.822 (0.769–0.882)	0.643 (0.455–0.856)	0.239 (0.196–0.294)
	Surgical Wards	0.928 (0.917–0.940)	0.909 (0.882–0.937)	0.819 (0.799–0.837)	0.603 (0.578–0.632)	0.969 (0.960–0.978)	0.092 (0.083–0.100)
	Non-Surg Med	0.851 (0.839–0.863)	0.882 (0.864–0.901)	0.645 (0.625–0.664)	0.584 (0.571–0.599)	0.906 (0.892–0.919)	0.154 (0.147–0.161)
XGBoost	Emergency Dept	0.973 (0.971–0.975)	0.825 (0.809–0.842)	0.952 (0.947–0.956)	0.772 (0.755–0.787)	0.965 (0.962–0.968)	0.051 (0.048–0.054)
	Critical Care	0.847 (0.730–0.945)	0.709 (0.582–0.821)	0.754 (0.572–0.936)	0.888 (0.808–0.964)	0.480 (0.365–0.599)	0.200 (0.140–0.269)
	Surgical Wards	0.939 (0.929–0.949)	0.777 (0.742–0.813)	0.909 (0.895–0.923)	0.721 (0.690–0.754)	0.931 (0.921–0.942)	0.089 (0.080–0.099)
	Non-Surg Med	0.872 (0.861–0.883)	0.739 (0.714–0.763)	0.823 (0.807–0.837)	0.702 (0.682–0.720)	0.848 (0.835–0.859)	0.150 (0.142–0.159)

Bold denotes the BIAlert sepsis model, the best-performing model in this evaluation.

## Data Availability

Data are available from the corresponding author upon reasonable request. Restrictions apply due to patient privacy.

## Abbreviations

The following abbreviations are used in this manuscript:

AI	Artificial Intelligence
AUC-ROC	Area Under the Receiver Operating Characteristic Curve
CEIC-Ib	Ethics and Health Research Committee of the Balearic Community
CI	Confidence Interval
CRP	C-Reactive Protein
ED	Emergency Department
eICU	eICU Collaborative Research Database
FiO_2_	Fraction of Inspired Oxygen
GCS	Glasgow Coma Scale
ICU	Intensive Care Unit
IIC	Instituto de Ingeniería del Conocimiento
LDH	Lactate Dehydrogenase
MAP	Mean Arterial Pressure
MIMIC	Medical Information Mart for Intensive Care
ML	Machine Learning
MSU	Multidisciplinary Sepsis Unit
NEWS	National Early Warning Score
NPV	Negative Predictive Value
qSOFA	quick Sequential Organ Failure Assessment
SD	Standard Deviation
SIRS	Systemic Inflammatory Response Syndrome
SOFA	Sequential Organ Failure Assessment
SVM	Support Vector Machine
TPE	Tree-structured Parzen Estimator
TREWS	Targeted Real-time Early Warning System
**XGBoost**	Extreme Gradient Boosting
